# The effect of acute social stress on the recognition of facial expression of emotions

**DOI:** 10.1038/s41598-017-01053-3

**Published:** 2017-04-21

**Authors:** Camille Daudelin-Peltier, Hélène Forget, Caroline Blais, Andréa Deschênes, Daniel Fiset

**Affiliations:** 1 Département de Psychoéducation et de Psychologie, Université du Québec en Outaouais, Gatineau, Canada; 2 Centre de Recherche en Neuropsychologie et Cognition, Montréal, Canada

## Abstract

This study investigates the effect of acute social stress on the recognition of facial expression of emotions in healthy young men. Participants underwent both a standardized psychosocial laboratory stressor (TSST-G) and a control condition. Then, they performed a homemade version of the facial expressions megamix. All six basic emotions were included in the task. First, our results show a systematic increase in the intensity threshold for disgust following stress, meaning that the participants’ performance with this emotion was impaired. We suggest that this may reflect an adaptive coping mechanism where participants attempt to decrease their anxiety and protect themselves from a socio-evaluative threat. Second, our results show a systematic decrease in the intensity threshold for surprise, therefore positively affecting the participants’ performance with that emotion. We suggest that the enhanced perception of surprise following the induction of social stress may be interpreted as an evolutionary adaptation, wherein being in a stressful environment increases the benefits of monitoring signals indicating the presence of a novel or threatening event. An alternative explanation may derive from the opposite nature of the facial expressions of disgust and surprise; the decreased recognition of disgust could therefore have fostered the propensity to perceive surprise.

## Introduction

In humans like in animals, the primary biobehavioral stress response has been characterized by the fight-or-flight response^1, 2^. Recent studies however suggest that humans might react differently when confronted to a social stress^3–5^. For instance, von Dawans and colleagues (2012) showed that individuals who experienced transitory acute psychosocial stress engaged in substantially more prosocial behaviour (trust, trustworthiness, and sharing) compared with participants who did not experience a socio-evaluative threat^5^. The authors explained their findings as manifestations of the human tendency to provide and receive joint protection within groups during threatening times^6, 7^. The human reaction to social stress therefore seems much more complex than the traditional fight-or-flight response. In this case, social stress appears to increase behaviour towards social proximity in humans, suggesting, in accordance with the tend-and-befriend hypothesis, a potent stress-buffering strategy^3, 4, 7^. Although initially proposed as characteristic of the female response^3^, some studies have recently revealed this pro-social response in men^5, 8, 9^.

From a neuroanatomical standpoint, the effect of stress on social behaviour is not surprising considering the relatively large body of evidence showing that brain areas frequently related to social behaviour, including the anterior cingulate cortex, the ventromedial prefrontal cortex and the amygdala^10, 11^ are targeted during an acute stress response^12–14^. Even though the aforementioned anatomical regions are also involved in basic functions of facial expression recognition^15–17^, to our knowledge, only one study has investigated the impact of an acute social stress on facial emotion recognition^18^. This study showed that healthy young boys (9 and 10 years old) who had experienced psychosocial stress were subsequently more likely to interpret ambiguous emotional expressions as fearful rather than angry (in the angry-fearful continuum). Although this study is very interesting, the limited number of tested facial expression continuums (i.e. happy-sad, happy-fearful, angry-fearful, and angry-sad) and the fact that it was conducted on young boys hinders the generalisability of its conclusions to adulthood. Moreover, since the main brain areas implicated in emotion processing, including the amygdala^19, 20^ and the PFC^21, 22^, continue to develop structurally throughout childhood and adolescence, it is easy to argue that stress could affect facial expression processing differently in adults. In accordance, some studies suggest that we could expect contrasting results in this population. For example, in healthy adults, it has been shown that a rise in cortisol induced by a psychosocial stress correlates with an increase in selective attention to socially threatening/rejecting faces (frowning faces)^23^ as well as with an enhanced processing of angry faces^24^. Hence, contrary to what was observed in 9 and 10 year old boys, psychological stress seems to induce a bias for anger in adult men.

Based on the foregoing evidence that stress may impact facial expression recognition in adults, the objective of the present study was to investigate the impact of an acute psychosocial stress on the recognition of facial expression of emotions in this population. In order to do so, we tested healthy young men on a homemade version of the facial expression megamix^25^ after they had been exposed to the Trier Social Stress Test for groups (TSST-G)^26^ or a control condition (see below for details).

## Methods

The Ethical Committee of the University du Québec en Outaouais (UQO) approved the present study and the experiment was performed in accordance with its relevant guidelines and regulations. Informed consent was obtained from all subjects for their participation.

### Participants

Volunteer male participants aged between 18 and 30 years old were recruited in the general population to participate in a study on social interactions. Only men were included in the study in order to avoid the well-known gender bias in emotional facial expression processing^27–32^ and given the potential confounding influence of female sex hormones on cortisol responses^33^. All participants were healthy Caucasians (with no neurological, psychiatric, endocrine, acute/chronic physical disease), right-handed and native French speakers with a normal or corrected-to-normal vision. None of them abused drugs or alcohol, nor smoked more than five cigarettes per day. Participants were naive to the Trier Social Stress Test (TSST) procedure and similar stress paradigms. In total, forty-two men participated in the present study. However, four subjects were excluded because the salivary samples used to verify their cortisol levels throughout the experiment did not show a cortisol increase in the stress condition and two were excluded because of a baseline cortisol level more than six standard deviations above the mean (mean = 6.44 nmol/l, S.D. = 3.26 nmol/l). The data presented here therefore includes thirty-six participants (mean age = 23.81 years old, S.D. = 3.87 years).

### Procedure

Subjects participated in groups of three. All investigations took place between 11:00 and 15:00 in order to minimize the influence of circadian variations in cortisol levels. Moreover, to ensure that saliva was not contaminated, participants were asked to refrain from drinking alcohol 24 hours before the experiment, from performing intense physical exercise 2 hours before the experiment and from brushing their teeth, eating, smoking and drinking anything else than water one hour before the experiment. Compliance with these restrictions was confirmed upon arrival. Prior to the experiment, all subjects completed an initial assessment of socio-demographic information. Participants were then submitted to one of the two experimental conditions (i.e. a psychosocial stress or a control condition; see description below). All subjects participated in both conditions in a counterbalanced order, so that half of the participants began with the stress condition and the other half, with the control condition. A within-subjects paradigm was chosen given individual differences in emotional facial expression processing^34, 35^ and the expected subtlety of the anticipated changes following the stress exposure. After being exposed to either condition, the participants completed the facial expression categorization task, i.e. the *Facial expression megamix*. The task was terminated after 30 minutes to ensure that the participants’ cortisol levels were significantly higher than the baseline level throughout the task (in the stress condition)^26, 36^. The two 75 minute experimental sessions took place in a laboratory at the UQO within a two-week range in order to minimize the influence of time on our participants’ performance. Immediately after the completion of the second experimental condition, participants were fully debriefed about the goal of the study and given insight into its nature. They also received a flat fee of 25 Canadian dollars for their participation.

#### Condition 1: stress induction

The Trier Social Stress Test for groups (TSST-G)^26^, an adapted version of the original TSST developed by Kirschbaum *et al*.^36^, was used to induce psychosocial stress. Similar to the TSST, the TSST-G is a standardized protocol that combines high levels of socio-evaluative threat (SET) and uncontrollability, but in a group format. The task consists of three phases: an anticipatory period (5 minutes), a public speaking task (3 minutes per participant) and a mental arithmetic task (3 minutes per participant). Like its original version, the TSST-G leads to a significant release of cortisol as a result of an activation of the hypothalamic-pituitary-adrenal (HPA) axis^26, 36^.

Upon their arrival, participants were seated individually and were not allowed to communicate with each other. They were then provided with written instructions for the TSST-G. More specifically, they were told that they had to perform two tasks in front of a selection committee and two video cameras: an oral task and a mental arithmetic task. The two judges of the panel, a man and a woman, were introduced as being trained in “behavioural observation” and participants were told that their overall performance would be recorded on video for subsequent analysis. In the first task, each participant had to introduce himself and give a three-minute speech in order to convince the selection committee of his suitability for a vacant position in a coveted job. Following the instructions, participants were given a paper and a pencil to help them organize their thoughts for the oral task (anticipation period). Their notes were not allowed during their speech. In the second task, participants had to serially subtract the number 13 from a previously assigned number – each participant received a distinct starting number in order to avoid learning effects. They were asked to count as quickly and as accurately as possible. On every failure, the committee asked the participant to start over. When transferred in the experimental room, participants were separated from each other by mobile walls in order to restrict any eye contact and social interaction.

Throughout the experiment, the two judges provided minimal feedback and displayed an emotionally neutral attitude. Participants were called in a random order to start their speech/mental arithmetic task.

#### Condition 2: control condition

The control condition of the TSST-G was designed to be as similar as possible to the TSST-G, excluding all stressing elements (i.e. committee, video cameras). This procedure was expected to eliminate the main effective factors of the TSST, namely the social-evaluative threat and the uncontrollability^37, 38^. Again, upon their arrival, participants were seated individually, were not allowed to communicate with each other and received written instructions for the control condition. Following the anticipatory period and their transfer to the experimental room, they were asked to silently read neutral magazine articles for nine minutes. They were then asked to count at a rate of “1” per second (starting from 0) for nine other minutes. The procedure took place in the same room as the TSST-G and the same mobile walls separated the participants from each other.

### Measures of stress response

To ensure that the stressful condition, in comparison with the control one, significantly increased stress in our sample of participants, seven salivary samples were collected at different moments (see below) during both experimental conditions. The participants were also asked to fill a questionnaire to measure their subjective stress level. Because this questionnaire takes between 2 and 5 minutes to fill, the subjective stress level was only measured twice: at the beginning and at the end of both experimental conditions.

#### Salivary collection and cortisol analysis

Recent studies have found that measuring salivary cortisol levels is a reliable biomarker of the physiological stress response^39^. Saliva was collected by asking the participants to chew on sterile cotton swabs for approximately one minute, until saturated. For biochemical analysis of free cortisol, salivary analyses were conducted in duplicate using ≪HS-cortisol High Sensitivity Salivary Cortisol≫ concentration. Saliva samples were thawed and spun at 3,000 revolutions per minute for 15 minutes to obtain a sufficient amount of clear saliva with low viscosity. The ≪Enzyme Immunoassay Kit≫ was purchased from Salimetrics, LLC (PA, USA). All salivary analyses took place at the UQO and were conducted by a laboratory technician. Inter- and intra-assay coefficients of variation were below 3.6% and 5.4%, respectively. Overall, seven saliva samples were collected during the experiment: at baseline (S1; −15 minutes), just before entering the experimental condition (S2; −1 minute), immediately after (S3; +18 minutes), and then four times at 10 minute intervals throughout the emotional task (S4; +28 minutes/S5; +38 minutes/S6; +48 minutes/S7; +58 minutes).

#### State Anxiety-Trait Inventory

To measure subjectively perceived levels of stress in experimental conditions, the French version^40^ of the State Anxiety subscale of the State Trait Anxiety Inventory (STAI-Y)^41^ was administered at the beginning and at the end of each experimental session. The STAI-Y is a commonly used measure of trait and state anxiety in both clinical and research settings, with higher scores being positively associated with higher levels of anxiety. The State Anxiety subscale consists of 20 questions concerning the subject’s current level of anxiety (eg. “I feel nervous”), that are meant to be answered on a four-point scale ranging from 1 (not at all) to 4 (extremely).

### Experimental task: Facial expression megamix

**Figure 1:**
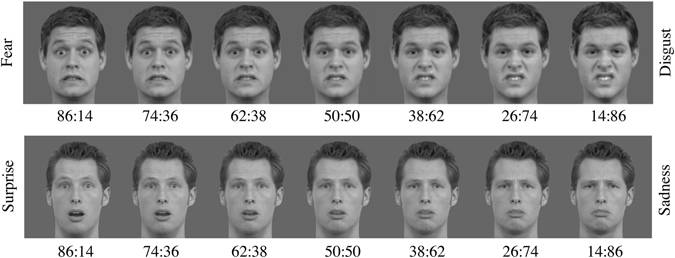
Examples of continua of morphed emotions used in the experimental task of Facial megamix expressions. The first continuum represents a combination of fear and disgust (identity 1) and the second continuum represents a combination of anger and sadness (identity 2).

The facial expression megamix is a very sensitive task evaluating the precise amount (in percentage) of an expression necessary for accurate recognition. The facial expression megamix paradigm used in our study was a homemade version of the original task, inspired by Young *et al*.^25^. Stimuli were taken from the *Karolinska Directed Emotional Faces database* ^42^. To avoid gender bias and since the stimuli used in the original facial expression megamix task were of series of facial expressions from the same man, a unique male identity was used for each experimental condition in a counterbalanced order^43^. All six basic emotions were included in the task (fear, anger, disgust, happiness, sadness and surprise) and were morphed with one another in all possible pairwise combinations using an image morphing software (*FantaMorph 5*.*0 for Mac*). The proportions of the facial expressions constituting each blend in a given continuum were 86:14, 74:26, 62:38, 50:50, 38:62, 26:74 and 14:86 (eg. 86% fear and 14% disgust would be one of the blends in the fear-disgust continuum). The prototypical expression (100%) was not used. Thus, for each identity, 105 morphed stimuli were generated (15 morphed facial expressions ×7 proportions) and used in this forced choice task. Prior to morphing, the pictures were touched up individually using *Photoshop*. Each one of them was converted to grayscale and placed on a neutral gray background (62.5 cd/m^2^). Image resolution was 256 × 256 pixels and the facial area subtended 6.2 degrees of visual angle. Viewing distance was maintained at 60 cm using a chinrest. The main inner facial features (eyes, nose, and mouth) were aligned within each stimulus set using rotation, translation, and scaling^44^. Finally, the stimuli were equated in mean luminance and spatial frequency spectrum with the SHINE toolbox^45^. Figure 1 provides examples of stimuli resulting from the combination of two emotions and used in the experimental task; more specifically, the fear-disgust (identity 1) and the surprise-sadness (identity 2) continua are illustrated.

Stimuli were presented one at the time in a random order at the center of a computer monitor and remained onscreen until response. The participants were asked to decide which prototypical expression the image most resembled. Responses were given using six labeled keys on the keyboard. No time limit was imposed to the participants and no feedback was given for the accuracy of the response. A training phase of five minutes (approximately 75 trials) preceded the task, allowing them to learn the appropriate key-emotion combinations. The results of the training phase were not included in the analyses. Participants were asked to perform a maximum of trials within 30 minutes, which resulted in approximately 450 trials per participant, per condition.

## Results

### Manipulation checks

**Figure 2:**
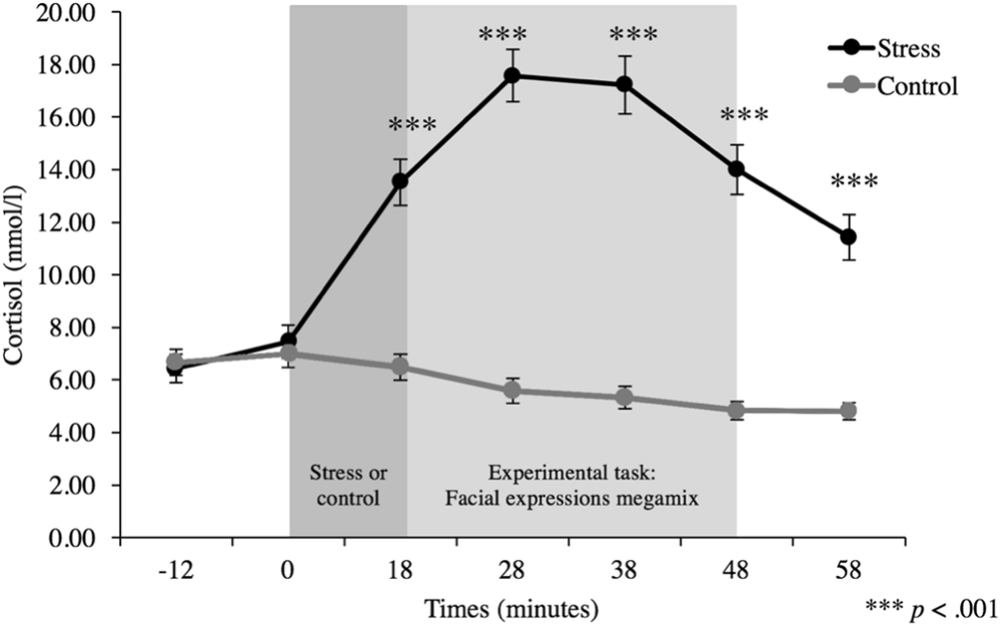
Comparison of participants’ salivary cortisol levels between experimental conditions.

To test whether psychosocial stress effectively induced an elevation in cortisol, we conducted a two-way (2 × 2 × 7) mixed ANOVA with experimental order (i.e. whether the participant performed the stress or the control condition first) as a between-subjects factor, and condition (i.e. stress or control) and time (i.e. the seven moments where the salivary samples were taken) as within-subjects factors. Experimental order was included as a between-subjects factor to ensure that the counterbalanced order of the experimental conditions had no impact on stress induction. Results show that the stress manipulation was successful. Significant results were found for the cortisol levels – main effect of condition [*F*(1, 34) = 115.01, *p* < 0.001, *η* ^2^ partial = 0.77] and time [*F*(2.27, 77.03) = 40.83, *p* < 0.001, *η* ^2^ partial = 0.55], as well as condition X time interaction [*F*(2.62, 88.92) = 94.25, *p* < 0.001, *η* ^2^ partial = 0.74]. Further exploring this interaction, we ran repeated-measure ANOVAs on the seven samples of cortisol level of each condition and revealed that, while the stress condition induced a significant increase in salivary free cortisol levels over time [*F*(2.43, 84.99) = 76.20, *p* < 0.001, *η* ^2^ partial = 0.69], a significant decrease in salivary free cortisol was observed over time in the control condition [*F*(2.27, 79.31) = 14.34, *p* < 0.001, *η* ^2^ partial = 0.29]. Single comparison follow-up analysis using paired t-tests specified that, in the stress condition, the level of cortisol remained significantly higher than in the control condition throughout the facial expression recognition task (samples 3 to 7, all at *p* < 0.001; two-sided, Bonferroni corrected). No significant difference in baseline cortisol [*t*(35) = −0.10, *p* = 0.93; two-sided] was found between the two conditions (see Fig. 2). It should be noted that the main effect of the experimental order factor [*F*(1, 34) = 0.94, *p* = 0.34], the experimental order X condition interaction [*F*(1, 34) = 1.13, *p* = 0.30], the experimental order X time interaction [*F*(2.27, 77.03) = 0.86, *p* = 0.44] and the experimental order X condition X time interaction were not significant [*F*(2.62, 88.92) = 1.33, *p* = 0.27], thus indicating that the activation of the HPA axis by the TSST-G was similar whether the participant started with the stress or the control condition.

**Figure 3:**
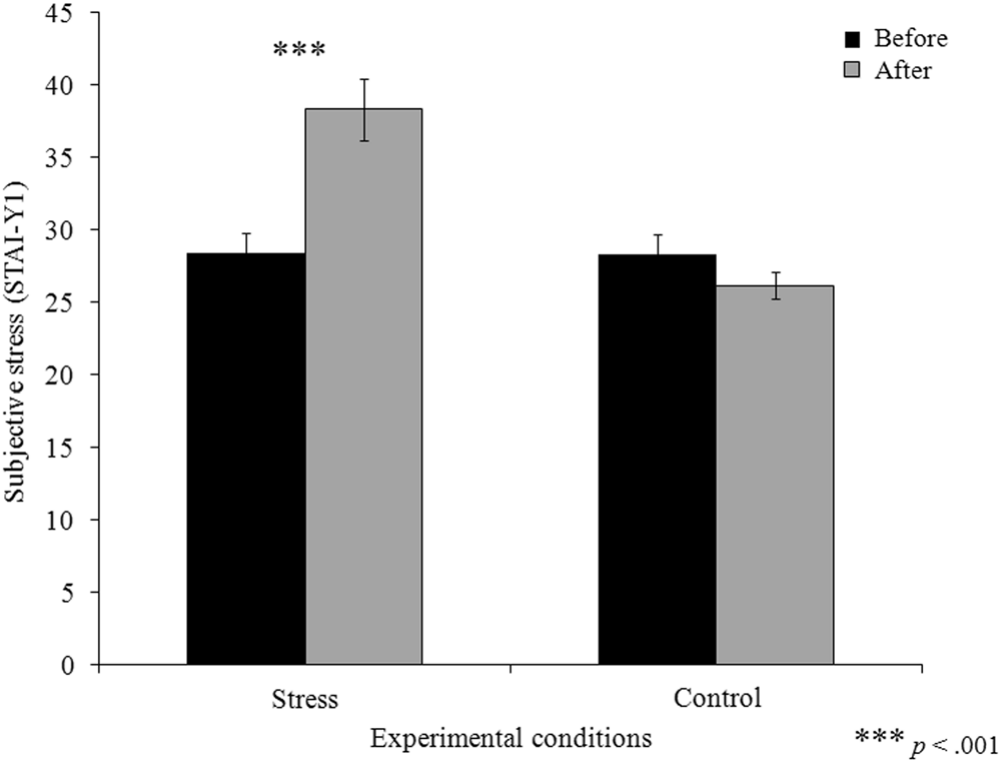
Comparison of participants’ subjective stress levels between experimental conditions.

We also tested whether the stress manipulation impacted subjective stress. To do so, we conducted a two-way (2 × 2 × 2) mixed ANOVA with experimental order (i.e. whether the participant performed the stress or the control condition first) as a between-subjects factor as well as condition (i.e. stress or control) and time (i.e. before and after the manipulations) as within-subjects factors. A main effect of condition [*F*(1, 34) = 33.38, *p* < 0.01, *η* ^2^ partial = 0.50] and time [*F*(1, 34) = 10.07, *p* < 0.01, *η* ^2^ partial = 0.23], as well as a condition X time interaction [*F*(1, 34) = 26.51, *p* < 0.001, *η* ^2^ partial = 0.44] reached significance. Single comparison follow-up analysis using paired t-tests confirmed that, in the stress condition, the participants’ subjective level of stress was significantly higher at the end compared to the beginning of the experiment [*t*(35) = −4.67, *p* < 0.001, *d* = 0.94; two-sided, Bonferroni corrected]. Conversely, in the control condition, the participants’ subjective stress level was significantly lower at the end than at the beginning of the experiment [*t*(35) = 2.30, *p* < 0.05, *d* = 0.31; two sided, Bonferroni corrected]. No significant difference was observed between the two conditions regarding the baseline level of subjective stress ratings [*t*(35) = 0.13, *p* = 0.89; two-sided] (see Fig. 3). Once again, it should be noted that the main effect of the experimental order factor [*F*(1, 34) = 2.05, *p* = 0.16], the experimental order X condition interaction [*F*(1, 34) = 1.30, *p* = 0.26], the experimental order X time interaction [*F*(1, 34) = 0.86, *p* = 0.36] and the experimental order X condition X time interaction were not significant [*F*(1, 34) = 0.02, *p* = 0.90]; this indicates that subjective stress was not affected by experimental order. Taken together, these results indicate that the experimental manipulation of stress induction was successful and was not affected by the counterbalanced order of the experimental sessions.

### Emotion recognition

#### Performance

To verify whether stress affected performance in facial expression recognition, we calculated the proportion of correct responses of each emotion. Answers were coded as correct when the expression perceived by the subject was represented with a proportion of at least 50% in the presented stimulus. For each expression, we pooled together all stimuli containing 62, 74 and 86% of a particular expression. A two-way mixed (2 × 2 × 6) ANOVA was conducted on the participants’ recognition accuracy, with experimental order as a between-subjects factor, as well as condition and emotion as within-subjects factors. Our results reveal a main effect of emotion [*F*(3.09, 104.98) = 37.94, *p* < 0.001, *η* ^2^ partial = 0.53], but the main effects of condition [*F*(1, 34) = 0.10, *p* = 0.77] and of experimental order [*F*(1, 34) = 0.07, *p* = 0.79] were not significant. The triple interaction experimental order X condition X emotion [*F*(2.45, 83.22) = 0.27, *p* = 0.81] was not significant. Crucially, the condition X emotion interaction [*F*(2.45, 83.22) = 3.82, *p* < 0.05, *η* ^2^ partial = 0.10] was significant. Single comparison follow-up analyses using paired t-tests showed that social stress impaired recognition accuracy for the expression of disgust [*t*(35) = −3.17, *p* < 0.05, *d* = 0.51; two- sided, Bonferroni corrected] and enhanced recognition accuracy for the expression of surprise [*t*(35) = 3.12, *p* < 0.05, *d* = 0.54; two- sided, Bonferroni corrected]. No significant difference was found for the four other expressions (all *p*’s > 0.14). The experimental order X emotion interaction [*F*(3.09, 104.98) = 0.35, *p* = 0.80] was not significant, but the experimental order X condition interaction [*F*(1, 34) = 4.18, *p* = 0.05] was marginally significant. This last result may suggest that the participants’ performance improved from the first to the second experimental session [*t*(35) = 2.03, *p* = 0.051, *d* = 0.24; two- sided, Bonferroni corrected]; nevertheless, it should be noted that since the condition x emotion interaction did not change as a function of the experimental order (i.e. triple interaction not significant), this situation does not affect the interpretation of our results.

#### Intensity threshold

**Figure 4:**
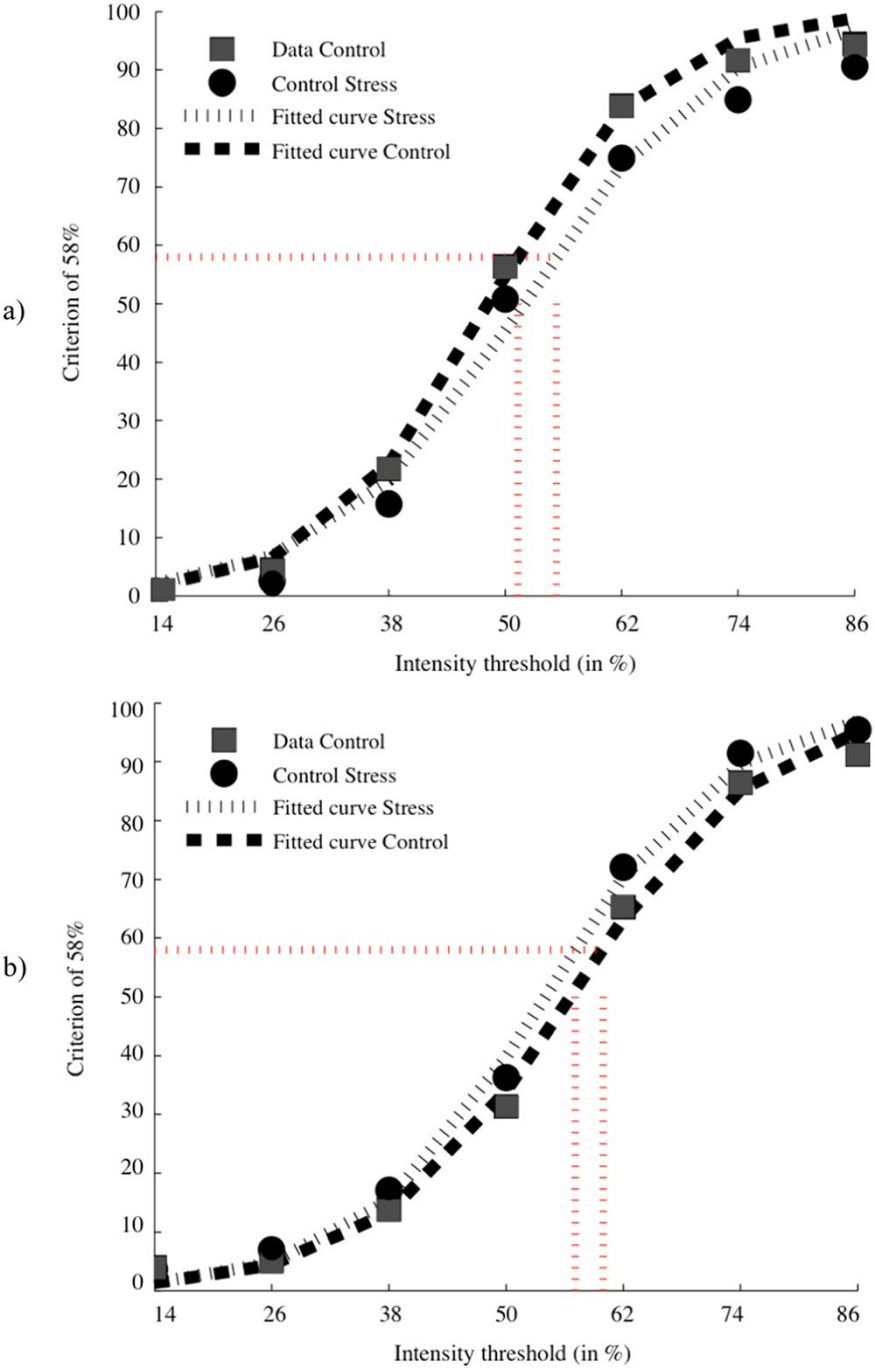
Intensity threshold between experimental conditions for (**a**) the expression of disgust and (**b**) the expression of surprise.

Since stress affected the participants’ performance for two facial expressions (i.e. disgust and surprise), we verified if this result could be explained by the impact of stress on the intensity threshold to which each expression was detected. To verify this hypothesis, we pooled together all continuums in which a given expression was represented and then calculated, for each level of the morph continuum (86:14, 74:26, 62:38, 50:50, 38:62, 26:74 and 14:86), the proportion of trials in which each expression was indicated as the subjects’ response. We then fit a sigmoid curve on these results to identify the required intensity threshold for each emotion to be perceived 58% of the time (in the continua where they were present). A criterion of 58% was established by identifying the midpoint between the percentage associated to chance (1/6 emotions = 16.67%) and the maximum performance (i.e. 100%). A two-way mixed (2 × 2 × 6) ANOVA was conducted on the participants’ recognition accuracy, with experimental order as a between-subjects factor as well as condition and time as within-subjects factors. This analysis revealed a significant effect of emotion [*F*(3.22, 109.43) = 25.83, *p* < 0.001, *η* ^2^ partial = 0.43], but the main effects of condition [*F*(1, 34) = 0.20, *p* = 0.66] and of experimental order [*F*(1, 34) = 0.21, *p* = 0.65] were not significant. The triple interaction experimental order X condition X emotion [*F*(2.27, 22.09) = 0.51, *p* = 0.63] was not significant. Crucially, the condition X emotion interaction [*F*(2.27, 77.09) = 3.28, *p* < 0.05, *η* ^2^ partial = 0.09] was significant. Single comparison follow-up analysis using paired t-tests showed that stress specifically increased the recognition threshold of the disgust expression [*t*(35) = 3.59, *p* < 0.01, *d* = 0.45; two-sided, Bonferroni corrected] and lowered the recognition threshold of the surprise expression [*t*(35) = −2.83, *p* < 0.05, *d* = 0.55; two-sided, Bonferroni corrected]. No significant difference was found for all four other expressions (all *p*’s > 0.13) (see Fig. 4). The experimental order X emotion interaction [*F*(3.22, 109.43) = 0.35, *p* = 0.88] was not significant, but the experimental order X condition interaction [*F*(1, 34) = 4.17, *p* = 0.05] was marginally significant. This last result may suggest that participants might have needed a lower intensity threshold for emotion recognition during the second experimental session in comparison to the first experimental session [*t*(35) = 2.03, *p* = 0.05, *d* = 0.25; two- sided, Bonferroni corrected]; however, as noted in the precedent section, the fact that the experimental order did not modulate the condition x emotion interaction indicates that the counterbalanced order did not influence the finding of different intensity thresholds with disgust and surprise as a function of stress.

#### Confusion

The results presented so far indicate that stress negatively affected the recognition of disgust, and conversely, positively modulated the recognition of surprise. Here, we verified if these results could be explained by a modification, following stress, in the pattern of confusions between disgust and the other emotions, and between surprise and the other emotions.

*Disgust*. For each condition, we calculated the proportion of responses in which each of the six emotions was given as an answer whenever disgust was dominant in the stimulus (i.e. when a morph containing 62% or more of disgust was presented). A two-way repeated measures ANOVA (2 × 6) was conducted on these proportions using the factors of condition and answered emotion. Results indicate a significant effect of answered emotion [*F*(1.23, 43.27) = 868.18, *p* < 0.001, *η* ^2^ partial = 0.96], but no effect of condition [*F*(1, 35) = 0.90, *p* = 0.77]. A significant effect was revealed for the condition X answered emotion interaction [*F*(1.23, 42.83) = 8.85, *p* < 0.05, *η* ^2^ partial = 0.20]. Single comparison follow-ups were conducted to reveal which emotions were most confused with disgust in the stress condition, compared to the control. These analyses indicate that when disgust was dominant in the stimulus, participants answered significantly less often the disgust expression [*t*(35) = −3.16, *p* < 0.05, *d* = 0.50; two-sided, Bonferroni corrected], while answering significantly more often the expression of anger [*t*(35) = 2.85, *p* < 0.05, *d* = 0.44; two-sided, Bonferroni corrected] and the happy expression [*t*(35) = 2.82, *p* < 0.05, *d* = 0.48; two-sided, Bonferroni corrected]. No difference was found for the four other emotions (all *p*’s > 0.64).

**Figure 5:**
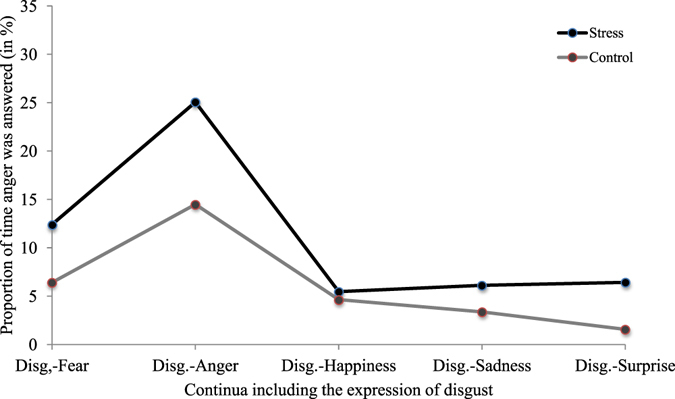
Proportion of time anger was answered when disgust was the dominant expression presented; for each combination of emotions including disgust and between experimental conditions.

We then verified if this tendency to confuse disgust with anger or happiness occurred no matter the expression mixed with disgust in the morphs, or only occurred specifically when anger or happiness were mixed with disgust. First, for all five continua including disgust (i.e. when disgust was mixed with anger, fear, happiness, sadness or surprise), we calculated the proportion of time, on average, that anger was given as an answer when disgust was the dominant expression. We submitted these proportions to a two-way repeated measures ANOVA (2 × 5) using the factors of condition and emotion presented. Results revealed a significant main effect of condition [*F*(1, 35) = 7.77, *p* < 0.01, *η* ^2^ partial = 0.18] and presented emotion [*F*(2.52, 88.49) = 36.07, *p* < 0.001, *η* ^2^ partial = 0.51], but no effect of the condition X emotion presented interaction [*F*(1.98, 69.32) = 3.05, *p* = 0.06]. These findings indicate that the increased perception of anger was apparent in all continua including disgust, although Fig. 5 illustrates that this general trend was less pronounced for the disgust-happy continuum. It is important to note, however, that the fact of categorizing more often the expression of anger was not a general bias (i.e. no matter the expression presented). In fact, as revealed by the ANOVAs on performance and intensity threshold, the general ability at recognizing anger did not change following an acute stress.

**Figure 6:**
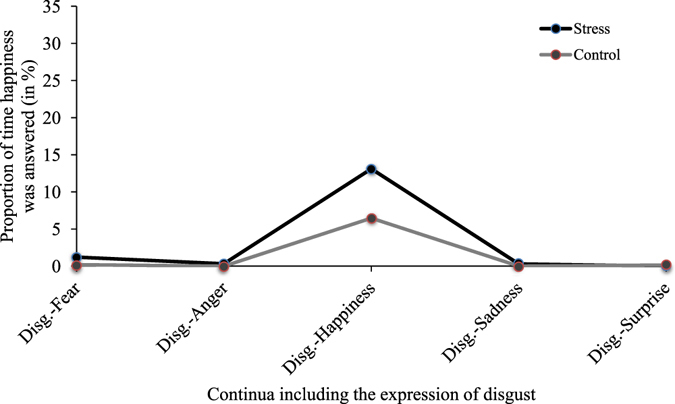
Proportion of time happiness was answered when disgust was the dominant expression presented; for each combination of emotions including disgust and between experimental conditions.

Similarly, for all five continua including disgust, we calculated the average proportion of times happy was given as an answer when disgust was the dominant expression presented. We submitted these proportions to a two-way repeated measures ANOVA (2 × 5) using the factors of condition and answered emotion. Results revealed a significant effect of condition [*F*(1, 35) = 7.74, *p* < 0.01, *η* ^2^ partial = 0.18], of answered emotion [*F*(1.04, 36.34) = 18.51, *p* < 0.001, *η* ^2^ partial = 0.35] and of the condition X answered emotion interaction [*F*(1.18, 41.22) = 5.83, *p* < 0.02, *η* ^2^ partial = 0.14]. Single comparison follow-up analyses using paired t-tests revealed that this confusion was specific to the disgust-happy continuum [*t*(35) = 2.59, *p* < 0.05, *d* = 0.43; two-sided, Bonferroni corrected] (all four other expressions at *p* > 0.17) (see Fig. 6).

Taken together, these analyses indicate that the impairment in the recognition of disgust following stress coincides with a tendency to categorize disgust as anger, except when the non-dominant emotion was happiness, in which case the dominant disgust expression was perceived as happiness. These confusions were specific to the five continua that included disgust; there was no change in happiness or anger recognition in continua that did not include the expression of disgust. This specificity confirms that the decreased sensitivity to disgust is likely the cause of the confusions.

**Figure 7:**
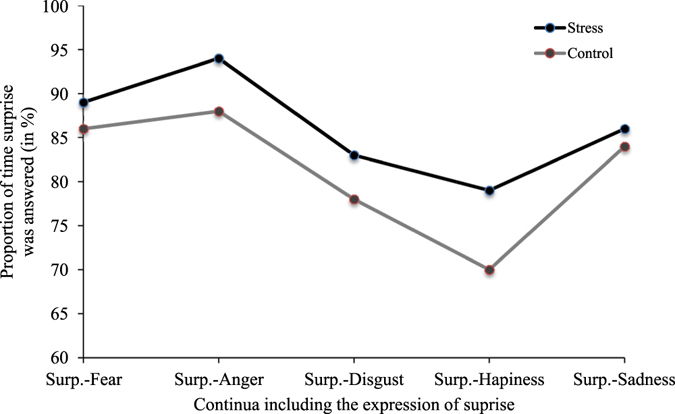
Proportion of time surprise was answered when it was the dominant expression presented; for each combination of emotions including surprise and between experimental conditions.

*Surprise*. We verified whether the increased recognition of surprise was explained by a tendency to answer significantly more often the expression of surprise in all continua including surprise or only in specific continua (eg. surprise-fear continuum). To do so, we calculated the average proportion of times surprise was given as an answer in all the continua in which it was included. We submitted these proportions to a two-way repeated measures ANOVA (2 × 5) using the factors of condition and presented emotion. Results revealed a significant main effect of condition [*F*(1, 35) = 9.78, *p* < 0.01, *η* ^2^ partial = 0.22] and presented emotion [*F*(4, 140) = 18.47, *p* < 0.001, *η* ^2^ partial = 0.51], but no effect of the condition X presented emotion interaction [*F*(2.95, 103.15) = 1.27, *p* = 0.29]. These findings indicate that the increased perception of surprise was apparent in all continua it which it was included (see Fig. 7).

We then sought to determine whether this tendency was restricted to continua including surprise or if it was also present in the other continua included in the experimental protocol. We thus calculated, for each condition, the average proportion of times that surprise was given as an answer in all continua included in the experimental protocol, except for the ones including surprise. We submitted these proportions to a two-way repeated measures ANOVA (2 × 5) using the factors of condition and answered emotion. Results revealed a significant effect of presented emotion [*F*(1.07, 37.36) = 61.15, *p* < 0.001, *η* ^2^ partial = 0.64] but no effect of condition [*F*(1, 35) = 0.04, *p* = 0.85], nor of the condition X emotion interaction [*F*(1.06, 37.22) = 0.05, *p* = 0.84]. This confirmed that, in the stress condition, the enhanced perception of surprise was specific to the continua in which it was included and was not a general trend found in all continua of the experimental protocol.

## Discussion

In this study, we investigated the impact of an acute psychosocial stress on the recognition of facial expressions of emotion. After being subjected to a stress or control condition, healthy young men performed the facial expression megamix task, which included all six basic emotions. Our physiological and subjective measures of stress were congruent: they both indicated a significant increase of stress in the stress condition and a significant decrease of stress in the control condition. In summary, our results show that psychosocial stress significantly decreases the recognition sensitivity of our participants to disgust, while significantly increasing their sensitivity to the expression of surprise. The participants’ performance with these emotions was affected accordingly.

### Disgust

As discussed in the Introduction, the tend-and-befriend conception stipulates that in a social stress context, human beings implement various strategies to promote social contact with their peers. This seems to occur in order to foster mutual joint protection^46^. A few recent studies have revealed this pro-social reaction –usually attributed to woman– among men; the results of the present study are congruent with this finding. Our results first show that in the stress condition, participants needed a higher percentage of the facial expression of disgust in order to recognize this emotion. We suggest that the inhibition of disgusted facial features follows the previous line of thought and reflects a perceptual mechanism involuntarily set up by the organism attempting to affiliate with and seek protection from its peers. Indeed, although the emotion of disgust was originally associated with the sense of taste and was described as an oral defense against something contaminated or repulsive^47–49^, its implications in our everyday life are now recognized as being much broader. In fact, its involvement in morality and in interpersonal experience is now increasingly studied^50, 51^. These studies suggest that interpersonal disgust (a concept close to contempt) strongly discourages social contact with other human beings who are not intimate. In this sense, one recent study showed that intimacy (ingroup relations) attenuates core disgust, which helps explain the ability of groups to cooperate^52^. The central point of the experience of disgust therefore includes the idea of “rejection”^51^. It is relevant to note that the insula, i.e. the main region involved in the experience and perception of disgust^53^, is also activated during an experience of social rejection^54, 55^. Similarly to social rejection, the socio-evaluative threat in the TSST paradigm includes the possibility of being negatively judged by others in a public context^56^. Overall, given the particular role of disgust in social rejection, it is easy to argue that the perception of the disgusted expression is not favourable to the promotion of social contacts with pairs (fear of rejection). Thus, the present finding of a decreased sensitivity to disgusted expressions may indicate that, within the threatening context of the TSST-G, stress led the participants to block the processing of disgusted facial expressions. In an everyday life situation, such a mechanism would provide a protection to individuals by allowing them to override the embarrassment of seeking the necessary support even from strangers and to reduce the situation-based anxiety. A similar explanation has been suggested in the context of psoriasis, a chronic stigmatizing skin disease that produces significant psychosocial distress and disability^57^. In their study, they found that psoriasis patients displayed a behavioural deficit as well as significantly smaller hemodynamic responses in the bilateral insular cortex when viewing disgusted facial expressions, but not for other negative emotions (i.e. fear and sadness). Psoriasis patients have long developed social anxiety and withdrawal due to social exclusion, and the mechanisms involved in the social anxiety experienced by these patients may differ from those involved in the social stress induced in a laboratory situation. Nevertheless, it is interesting to note that both situations involve an experience of social anxiety, and they are both linked with a decreased perception of disgust.

Although speculative, this proposal is consistent with our data as well as with the current literature. As mentioned earlier, a recent study has investigated the effect of acute social stress on facial expression recognition in a population of young boys^18^. The results showed that, following acute social stress, young boys displayed a tendency to interpret ambiguous emotional expressions as less angry and more fearful (in the angry-fearful continuum). The authors suggested that the decreased perception of anger might reflect an adaptive coping mechanism aiming at encouraging social approach behaviour. The discrepancy between the results obtained with young boys and adult males may be explained by methodological differences (i.e. the study by Chen *et al*. did not include the emotions of disgust and surprise) or by developmental differences (i.e. the main brain areas implicated in emotion processing continue to develop structurally throughout childhood and adolescence). However, the hypothesis of an adaptive coping mechanism may also be consistent with the one we proposed above explaining the decreased perception of disgust in adult males, if one assumes that the nature of the threat experienced during the TSST-G modulates emotion perception. More specifically, in both studies, participants underwent the TSST-G in front of adult judges and then viewed adult faces in the experimental task. The difference between these studies is that while participants of our study were confronted to peer faces (faces of their age group), the young boys included in Chen *et al*.’s study were rather confronted to authority figures (adults). It is therefore very possible that the threat experienced by our adult participants was the negative judgment/rejection from peers (leading to the inhibition of disgust – the emotion associated to social rejection), while the main fear experienced by those children was to be reprimanded. Since angry is undeniably a facial expression reflecting dominance^58–60^, the results obtained by Chen *et al*. may have been the consequence of a protective mechanism implemented by children to inhibit the facial features of that expression^19^. In a future study, it would be very interesting to verify if these results would be different if the stimuli viewed by children were peer faces (i.e. faces of their age group).

We also performed an analysis on the errors made by our participants in the stress condition. This analysis showed that the inhibition of disgust had perceptual consequences on other facial expressions; this analysis revealed two specific patterns of errors. The first pattern of errors displayed by stressed participants concerns their propensity to respond that they perceived anger when disgust was the dominant expression. The confusion between anger and disgust is consistent with the fact that these two emotions share facial signals, and may be confused with one another when wrong visual information is processed^61^. Thus it is strongly possible that the perceptual inhibition of the visual information associated with disgust led the participants to process information rather related to anger, causing them to miscategorise disgust as anger. The fact that this pattern of errors was not found in the continua where disgust was not present, and the fact that stress had no significant impact on the recognition threshold of anger also corroborates this interpretation, and excludes the possibility of a response bias toward anger, or of a targeted change in the perception of the this expression. It is important to note that despite the perceptual confusion between disgust and anger found in our results, this does not suggest that stress simply amplified susceptibility to existing perceptual similarities between the two emotions. If so, stress would have predicted other types of frequent confusions (eg. fear-surprise, sadness-fear) that were not observed^62, 63^. It rather created confusions not only based on the visual similarity of facial expressions, but also on a higher order. In this sense, the confusion between anger and disgust could also be explained by their underlying dimensions of “valence” and “arousal”. One of the dominant theories in the field of facial emotion is the dimensional theory^64^. This theory posits the existence of two fundamental dimensions of emotional space: valence and arousal. Valence represents the hedonic position on a pleasantness–unpleasantness continuum, whereas arousal refers to the level of intensity of an affective experience. Although anger and disgust emotions are both of negative valence, the former is associated with a slightly higher level of arousal than the latter. As social stress induces a state of higher arousal, it is possible that it led the participants to be more sensitive to anger at the expense of disgust^65^.

The second pattern of errors displayed by stressed participants concerns the disgust-happy continuum specifically. For this particular continuum, participants responded more frequently using the happy expression. In this case, it is very likely that the perceptual saliency of happiness countered the perception of the disgust/anger expression, leading participants to respond using this emotion in the presence of this specific continuum. This hypothesis seems even more plausible given that happiness practically shares no facial features with anger^48, 66^.

Furthermore, it seems markedly relevant to discuss the parallel between our results and those obtained in studies investigating facial expression recognition deficiencies in people suffering from social anxiety. Patients with social anxiety have been found to be significantly less sensitive, and therefore perform significantly worse, with negative emotions, particularly with disgust and anger^67^. In a manner similar to ours, the authors explained their findings by suggesting that people with social anxiety inhibit these negative emotions in order to reduce experienced anxiety. In contrast with these results, we found no evidence of an impaired recognition of anger in subjects exposed to acute psychosocial stress. This difference can probably be explained by the sudden and socio-evaluative nature of the TSST-G. Indeed, it is unlikely that our participants experienced a danger other than being judged negatively. In the same vein, they weren’t driven by the core beliefs of imminent threat and personal vulnerability that develop through repeated experiences of pathological anxiety, which could explain why we found no difference regarding anger. Nonetheless, more research is needed to compare the similarities and differences in the mechanisms and strategies used by socially anxious patients and healthy participants that experience momentary stress as it is the case during the TSST-G.

### Surprise

Unlike what happened with disgust, the social stress experienced by our participants significantly improved their performance with surprise. More precisely, our results suggest a systematic decrease in the intensity threshold necessary for recognizing this emotion following exposure to stress. A more thorough analysis showed that the increased perception of surprise did not interact with the facial expression with which the morph was created.

Previous studies have shown that surprise is a powerful emotional signal when it comes to directing the attention towards a potentially novel or threatening event^68–70^. In fact, when a face displaying an averted gaze is presented to participants, they typically move their attention in the direction indicated by the gaze^71–73^, a finding called the gaze orienting effect. This effect is even stronger when the face displays a fearful or surprised expression, and, interestingly, is as strong with surprise as it is with fear^68–70^. The impact of fearful and surprised expressions on the gaze orienting effect has been interpreted as an evolutionary adaptation, since rapidly orienting gaze towards an unexpected or potentially threatening situation is beneficial for survival. The present finding of an increased perception of surprise following the induction of a social stress may also be linked to such an evolutionary adaptation, wherein being in a stressful environment increases the perceived benefits of monitoring signals indicating the presence of a novel or threatening event. Moreover, although surprised expressions involve the activation of action units overlapping only with fearful expressions, the latter involve the activation of action unit 4, which is also present in the expression of anger^74^. In the present context, where pairs of expressions were mixed together, the overlap in the action unit involved in anger and fear may have rendered the signals contained in fearful expressions less distinctive indicators of novelty than the ones that make up the expression of surprise.

An alternative hypothesis to explain the results lies in the observation that disgust and surprise have frequently been opposed in models attempting to better understand and explain the existing nuances, relationships and confusions between the different facial expressions of emotions. For example, the bidimensional model of Woodworth & Schlosberg^75^ opposes these two emotions on an imaginary circle in which the two orthogonal dimensions correspond to pleasant-unpleasant and attention-rejection^75^. Similarly, the multidimensional perceptual model suggested by Young and al.^25^ position disgust and surprise on opposite corners of an hexagonal representation of the relations between emotions^25^. More recently, a cubic model developed by Lövheim^76^ proposed a direct relation between specific combinations of predefined levels of monoamine neurotransmitters (serotonin, dopamine and norepinephrine) and certain basic emotions^76^. Once again, this model opposed the recognition of disgust and surprise based on the level of norepinephrine, a very important hormone implicated in the stress response (their level of serotonin and dopamine being the same). Although more empirical research on the opposite nature of disgust and surprise is certainly needed, it is interesting to consider the idea that the decreased recognition of disgust fostered the propensity to perceive surprise.

In a future investigation, it would be interesting to replicate the paradigm used in the present study while including morphs of each expression with a neutral expression. This would help establish whether there are different detection thresholds for each expression as a function of the stress condition, since theoretically the neutral expression represents the center point of the multidimensional Woodworth and Schlosberg model^75^. This might, in addition, contribute to clarify which of the previous hypothesis regarding the recognition of surprise explains in greater part the enhanced visual sensitivity to that emotion.

## Conclusion

In summary, our results showed that acute psychological stress negatively affected the perception and recognition of disgust. More specifically, the perceptual inhibition of the disgusted facial expression led participants to miscategorise this emotion as anger, possibly due to the proximity of these emotions in terms of visual cues (except for the disgust-happy continuum where happy was more frequently given as an answer). Our results also showed that acute psychological stress positively affected the recognition of surprise. The increased perception of surprise was present in all continua in which this emotion was included. Overall, our study clearly highlights the neglected role of disgust, and possibly surprise, in social stress experiences. Our data highlights the importance of including these two facial expressions in future investigations concerning the impact of stress and social anxiety on the recognition of facial expressions of emotion. So far, the majority of studies investigating the impact of stress or social anxiety on several emotional components only included one positive expression (usually happiness), one negative expression (usually anger) and one neutral expression. This might explain why most researchers initially assume that anger is the most appropriate emotion in relation to social concerns. Notably, recent research suggests that disgust plays a more prominent role than anger in social anxiety^77–79^ since it conveys potential harm, namely disapproval and rejection by peers, which are categorically avoided by people experiencing symptoms of that psychological disorder^80^. Our study supports this idea and offers a novel perspective on the distinctive social role of disgust that has, until recently, been overlooked.
